# Protein tyrosine phosphatase 1B targets PITX1/p120RasGAP thus showing therapeutic potential in colorectal carcinoma

**DOI:** 10.1038/srep35308

**Published:** 2016-10-18

**Authors:** Hao-Wei Teng, Man-Hsin Hung, Li-Ju Chen, Mao-Ju Chang, Feng-Shu Hsieh, Ming-Hsien Tsai, Jui-Wen Huang, Chih-Lung Lin, Hsiang-Wen Tseng, Zong-Keng Kuo, Jeng-Kai Jiang, Shung-Haur Yang, Chung-Wai Shiau, Kuen-Feng Chen

**Affiliations:** 1 Department of Medical Research, National Taiwan University Hospital, Taipei, Taiwan; 2 National Center of Excellence for Clinical Trial and Research, National Taiwan University Hospital, Taipei, Taiwan; 3 Division of Medical Oncology, Department of Oncology, Taipei Veterans General Hospital, Taipei, Taiwan; 4 School of Medicine, National Yang-Ming University, Taipei, Taiwan; 5 Program in Molecular Medicine, School of Life Science, National Yang-Ming University, Taipei, Taiwan; 6 Industrial Technology Research Institute, Hsin-Chu, Taiwan; 7 Division of Colon & Rectal Surgery, Department of Surgery, Taipei Veterans General Hospital, Taipei, Taiwan; 8 Institute of Biopharmaceutical Sciences, National Yang-Ming University, Taipei, Taiwan

## Abstract

Protein tyrosine phosphatase 1B (PTP1B) is known to promote the pathogenesis of diabetes and obesity by negatively regulating insulin and leptin pathways, but its role associated with colon carcinogenesis is still under debate. In this study, we demonstrated the oncogenic role of PTP1B in promoting colon carcinogenesis and predicting worse clinical outcomes in CRC patients. By co-immunoprecipitation, we showed that PITX1 was a novel substrate of PTP1B. Through direct dephosphorylation at Y160, Y175 and Y179, PTP1B destabilized PITX1, which resulted in downregulation of the PITX1/p120RasGAP axis. Interestingly, we found that regorafenib, the approved target agent for advanced CRC patients, exerted a novel property against PTP1B. By inhibiting PTP1B activity, regorafenib treatment augmented the stability of PITX1 protein and upregulated the expression of p120RasGAP in CRC. Importantly, we found that this PTP1B-dependant PITX1/p120RasGAP axis determines the *in vitro* anti-CRC effects of regorafenib. The above-mentioned effects of regorafenib were confirmed by the HT-29 xenograft tumor model. In conclusion, we demonstrated a novel oncogenic mechanism of PTP1B on affecting PITX1/p120RasGAP in CRC. Regorafenib inhibited CRC survival through reserving PTP1B-dependant PITX1/p120RasGAP downregulation. PTP1B may be a potential biomarker predicting regorafenib effectiveness, and a potential solution for CRC.

Colorectal carcinoma (CRC) is the third most common cancer in the world, with more than 1.3 million new cases diagnosed annually^1^. Interestingly, there is a large geographic difference in the worldwide distribution of CRC; the difference of incidence between countries with the highest and the lowest rates varying up to 10-fold^2^. A canonical multistep process involving sequential genetic mutations in APC, K-ras and p53 has been established to explain colon carcinogenesis^3^; however, the global heterogeneity in CRC incidence indicates that such a model is not fit to explain all CRC patients. Thus, it is important to investigate potential oncogenic factors associated with the development of CRC, especially those that may link tumor biology to known environmental and lifestyle risk factors, such as obesity^4^ and insulin^5^,^6^ resistance of CRC.

Protein tyrosine phosphatase 1B (PTP1B, *PTPN1*), the prototypic member of the protein tyrosine phosphatase superfamily, has been implicated in multiple signaling pathways associated with cell growth and metabolism^7^,^8^,^9^. PTP1B is known to attenuate signaling from insulin and leptin receptors, and, thus, play a major role in the pathogenesis of type 2 diabetes and obesity^10^,^11^,^12^. Distinct from the clear functions characterized in metabolic regulation, the effects of PTP1B on carcinogenesis are far more complex. PTP1B was originally considered to play a tumor suppressor role counteracting activated tyrosine kinases, such as Bcr-Abl, β-catenin, and epithelial growth factor receptor^13^,^14^,^15^; however, a growing body of evidence also supports the notion that PTP1B is actively involved in promoting carcinogenesis by interacting with several oncogenic substrates, including HER2/*Neu*, ERK1/2, p62Dok and Src^8^,^16^,^17^. The seemingly contradictory effects of PTP1B on oncogenesis reflect the complex interplay of PTP1B-regulated signaling and the diverged oncogenic addiction on metabolism and growth signaling in each cancer type. For CRC, Zhu *et al*. described a positive role for PTP1B in the promotion of colon carcinogenesis through Src Activation^17^, and overexpression of PTP1B was found to be an important prognostic factor in CRC patients^18^,^19^. Intriguingly, Tremblay and colleagues suggested that PTP1B has a positive regulatory role in Ras activity by finding that upregulation of p120 Ras GTPase-activating protein (p120RasGAP, RASA1) and p62Dok phosphorylation were presented in PTP1B-deficient fibroblasts^8^. Given the crucial role of Ras signaling in insulin receptor signaling^9^,^20^ and colon carcinogenesis^3^,^21^, it is of great interest to explore the cross talk between PTP1B and Ras signaling in CRC.

In the current work, we describe a novel oncogenic mechanism of PTP1B in CRC through affecting the PITX-1/p120RasGAP axis. By dephosphorylating PITX-1, the transcription factor of p120RasGAP^22^, PTP1B destabilizes PITX-1 protein and downregulates p120RasGAP to promote proliferation of CRC cells. Furthermore, we show that the expression of PTP1B in clinical CRC tumors is negatively correlated with p120RasGAP, but positive correlated with p-Erk expression, one indicator for activation of Ras signaling. Overexpression of PTP1B predicts early development of metastasis and shorter overall survival in CRC patients. Moreover, we further identified that regorafenib, a multi-kinase inhibitor that has been approved for chemo-refractory metastatic CRC patients^23^, modulates PTP1B/PITX-1/p120RasGAP signaling in CRC.

## Results

### PTP1B is a novel oncoprotein in colorectal carcinoma

**Figure 1:**
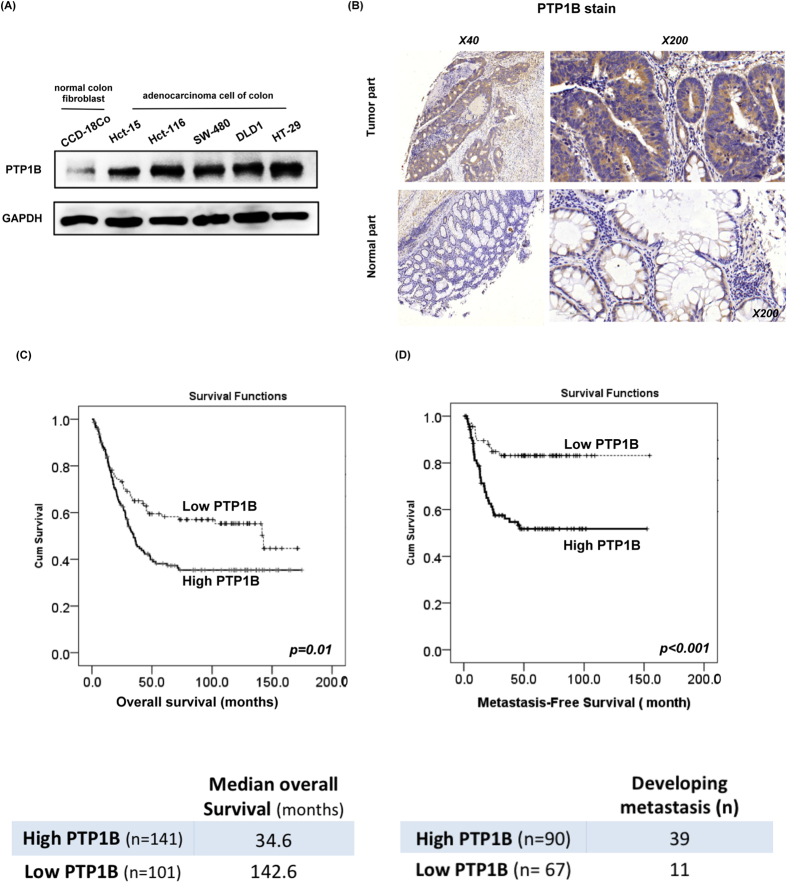
PTP1B is a novel oncoprotein in human colorectal cancer. (**A**) PTP1B is highly expressed in CRC. The endogenous expression levels of PTP1B in 5 CRC cell lines (Hct-15, Hct-116, SW-480, DLD1 and Ht-29) and a normal colon fibroblast cell line (CCD-18Co) were determined by western blot (upper panel). GAPDH was used as a loading control. (**B**) Representative immunohistochemical staining of PTP1B in clinical samples. A cohort of 242 patients with CRC was examined and the expression of PTP1B was much higher in the tumor part than the adjacent normal part. (**C**) Overall survival plot of 242 CRC patients after CRC diagnosis. High expression of PTP1B was associated with worse overall survival in CRC patients (p = 0.01). (**D**) Metastatic-free survival of 157 CRC patients with stage I-III disease at diagnosis. High expression of PTP1B was associated with worse metastatic-free survival in CRC patients (p < 0.001).

To understand the role of PTP1B in CRC, we first examined the expression of PTP1B in a panel of cell lines containing CRC and normal colon cells. As shown in Fig. 1A, PTP1B was highly expressed in all CRC cell lines, but not in the CCD-18Co cells (normal fibroblasts from the colon). Interestingly, we found very similar results when analyzing clinical samples obtained from 242 CRC patients, whose basic characteristics are summarized in Supplement Table 1. The expression of PTP1B was higher in the tumor tissue compared to the adjacent normal part (Fig. 1B). Moreover, patients with overexpression of PTP1B had more aggressive clinical presentation. Within a median 46 months follow-up period, 43.3% patients with high PTP1B expression had newly-identified metastatic disease, while it was 16.4% in patients with low PTP1B expression. Using Kaplan-Meier survival analysis, we found that overexpression of PTP1B significantly defined a subgroup of patients with worse overall survival (*P* = 0.01, Fig. 1C) and metastatic–free survival (*P* < 0.001, Fig. 1D).

**Figure 2:**
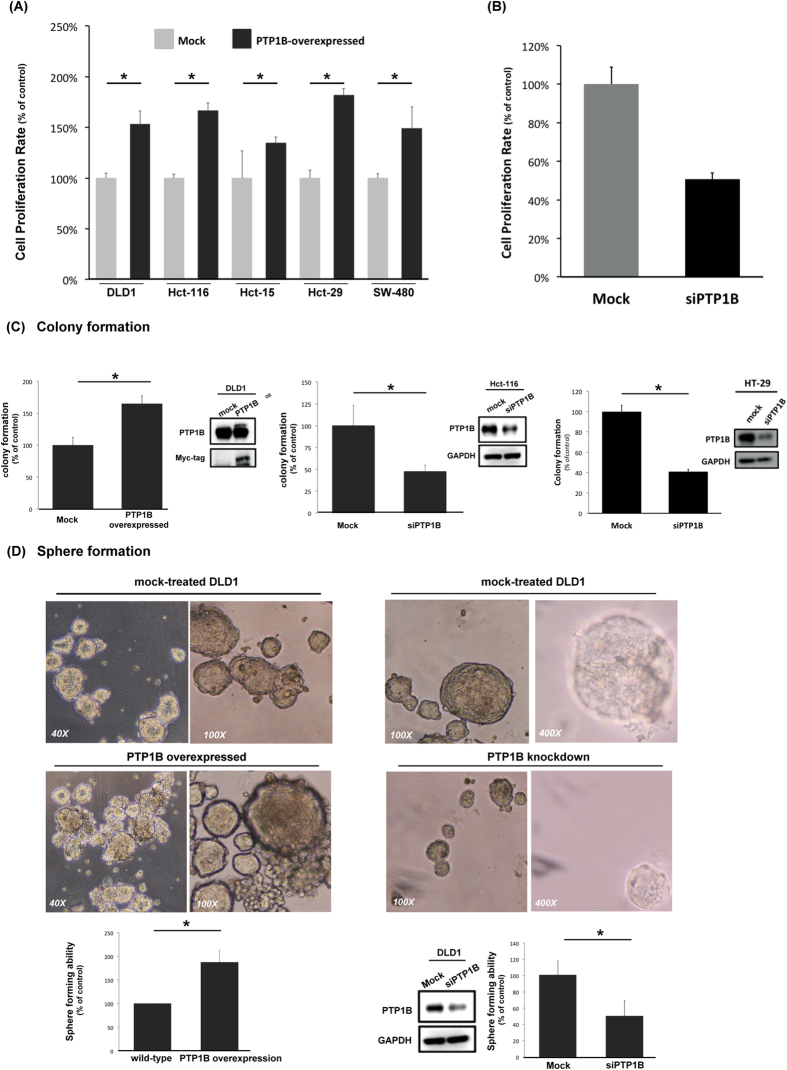
PTP1B expression is associated with the tumorigenic properties of CRC. (**A**) Overexpression of PTP1B potentiated the proliferation rate of CRC cells. Cell proliferation of CRC cancer cells with and without overexpression of PTP1B was assessed by MTT assay. Cells were transfected with vector or PTP1B for 24 hours and then reseeded in a 96-well plate for another 72 hours. Columns, mean; bars, SD (*n* = 4). *P < 0.05; **P < 0.01. (**B**) Knockdown of PTP1B significantly reduced the proliferation rate of Hct-29 cell. The viability of Hct-29 was determined by MTT. Columns, mean; bars, SD (*n* = 3). (**C,D**) PTP1B expression was significantly associated with the tumorigenicity of CRC. Colony formation assay (**C**) and sphere formation assay (**D**) were used to determine the ability of CRC formation. For colon colonies, indicated cells were seeded in 10-cm dishes and analyzed after two weeks. For sphere formation assay, DLD1 cells transfected with PTP1B or siPTP1B were seeded in ultra-low attachment 24-well plate and f grew in serum-free median for14 days. The results of the above-mentioned tests were quantified. Columns, mean; bars, SD (*n* = 3). *P < 0.05; **P < 0.01.

Next, we sought to understand the impact of PTP1B on promotion of carcinogenesis in CRC using three different assays. First, by MTT, we found that overexpression of PTP1B significantly increased the proliferation rates of all CRC cells, while PTP1B silencing resulted in reduced proliferation of HCT-29 cell (Fig. 2A,B). Correspondingly, the number of colonies formed by DLD1 cells with ectopic expression of PTP1B was nearly two times more than that of mock-treated cells, while PTP1B-knockdown Hct116 and Hct29 cells generated fewer colonies than the control (Fig. 2C). Last, we performed sphere formation assay and found that overexpression of PTP1B significantly potentiated the sphere forming ability of DLD1 cells, while knockdown of PTP1b reduced the number and size of CRC spheres formed by DLD1 and Hct29 cells (Fig. 2D and supplement Figure 1). Together these results show that overexpression of PTP1B is a recurrent event in CRC patients and was associated with worse clinical outcome. Moreover, PTP1B expression was critically associated with the carcinogenic properties of CRC cells.

### PTP1B affects the PITX1/RasGAP axis in CRC cells

**Figure 3:**
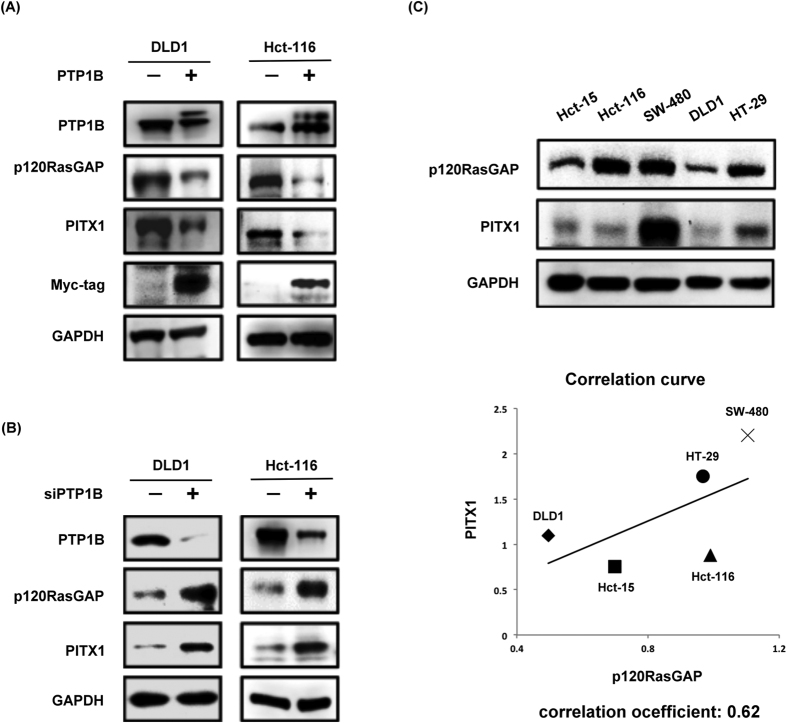
PTP1B affects PITX-1/RasGAP in CRC. (**A**) Overexpression of PTP1B resulted in downregulation of PITX1 and p120RasGAP. DLD1 and Hct-116 cells with and without PTP1B ectopic overexpression were harvested and analyzed by western blot. Representative image of western blot were shown (N = 3). (**B**) Siliencing of PTP1B leaded to augmentation of PITX1 and p120RasGAP expression in CRC cells. DLD1 and Hct-116 cells with and without knockdown of PTP1B were harvested and analyzed by western blot. Representative image of western blot were shown (N = 3). (**C**) Expression of PITX1 and p120RasGAP were highly correlated in CRC cells. Expression of PITX1 and p120RasGAP in CRC cells were examined and quantified. Represensative western blot images were shown in upper panel (N = 3). The correlation of protein expression was determined by linear regression (lower panel).

To explore the mechanism by which PTP1B promotes carcinogenesis, we examined the biological impact of PTP1B-overexpression in CRC cells. Interestingly, we found that overexpression of PTP1B in DLD1 and Hct-116 cells significantly downregulated the expression of p120RasGAP and Pitx1 (Fig. 3A). To confirm this finding, we knocked down PTP1B in CRC cells, and, indeed, the expression of p120RasGAP and PITX-1 was upregulated (Fig. 3B). According to the literature^22^, PITX-1 is involved in regulating the transcriptional activity of RasGAP. Thus, we sought to validate this relationship by examining the endogenous expression of PITX-1 and p120RasGAP in CRC cells. As shown in Fig. 3C, the endogenous expression of these two proteins were highly correlated. Therefore, we postulated that PTP1B induced downregulation of p120RasGAP via affecting PITX1 and inhibition of the PITX-1/RasGAP axis mediates the oncogenic effects of PTP1B in CRC.

### PTP1B downregulates the expression of PITX1 through affecting phospho(Y)-PITX1

**Figure 4:**
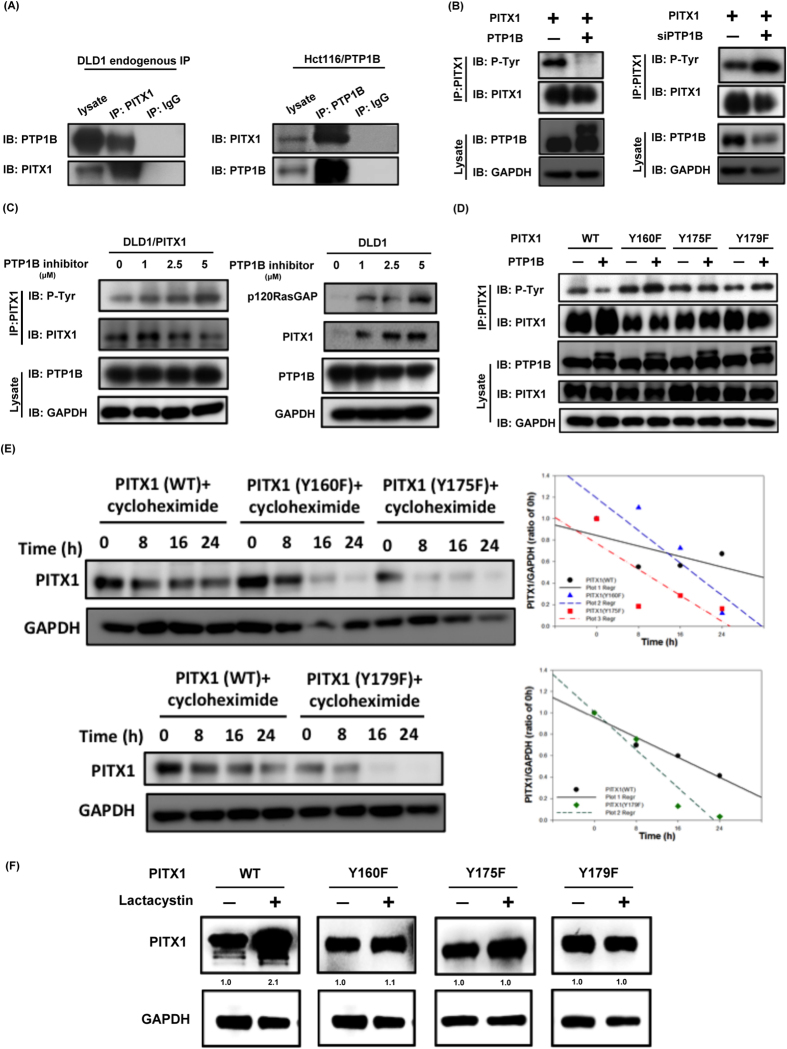
PTP1B directly dephosphorylates PITX1, which leads to destabilization of PITX1 protein. (**A**) PTP1B directly associated with PITX-1. The interactions between PITX-1 and PTP1B were detected in DLD1 endogenous immunoprecipitation (left panel) and Hct-116 overexpression with PTP1B immunoprecipitation (right panel). Representative images of triplicated experiments were shown here. (**B**) Modulation of PTP1B levels influenced the phosphorylation status of endogenous PITX-1 in DLD1 cells. Ectopic expression of PTP1B induced tyrosine dephosphorylation of PITX-1 (left panel) and silencing of PTP1B upregulated the phospho-PITX-1 level (right panel). Representative images of triplicated experiments were shown here. (**C**) Specific PTP1B inhibitor treatment increased the phospho-tyrosine status of PITX1 and augmented PITX1/p120RasGAP expression in CRC cells. The phospho-tyrosine status of PITX1 in PITX1-overexpressed DLD1 cells were determined by immunoprecipitation assay after exposing to PTP1B inhibitor treatment at the indicated doses for 4 hours (left panel). The expression of PITX1/p120RasGAP after PTP1B treatment described above were also examined by western blot (right panel). Representative images of triplicated experiments were shown here. (**D**) PTP1B interacted with PITX-1 at three tyrosine residues, namely Y160, Y175 and Y179. PTP1B could only induced PITX-1 phosphorylation change in wild-type PITX-1-expressing cells. Phospho-(T)-PITX1 in DLD1 expressing wild-type, Y160F-, Y175F-, and Y79F-mutant PITX1 were determined by immuneprecipitation and immunebloting. Representative images of triplicated experiments were shown here. (**E**) PTP1B-dependent dephosphorylation reduced the stability of PITX-1. DLD1 cells with different PITX-1-phospho-mutants were treated with 50 μg/ml cycloheximide (CHX) for the indicated times and analyzed by western blot. Representative images of triplicated experiments were shown over the left panel. The quantified results of PITX1 expression in each experiment were shown over the right panel (n = 3). (**F**) Degradation of wild-type and mutant PITX-1 protein were affected by lactacystin treatment. Effects of proteasome inhibitor, Lacacystin, on wild-type or the three mutants PITX-1 were examined by western blot (N = 3).

Next, we examined whether and how PTP1B affects the expression of PITX1/p120RasGAP through PITX1. Using endogenous and overexpression co-immunoprecipitation assay, a direct association between PITX-1 and PTP1B was identified (Fig. 4A and Supplement Figure 2A). Since PTP1B is known to be a tyrosine phosphatase, we postulated that PTP1B affects the expression of PITX-1 by dephosphorylation. To prove our hypothesis, we manipulated the expression level of PTP1B and examined the phospho(Y)-status of PITX1. In DLD1 cell with ectopic expression of PTP1B, we found that the phospho(Y)-PTIX1 was downregulated; and conversely, silencing of PTP1B resulted in upreguation of phospho(Y)-PITX1 (Fig. 4B and Supplement Figure 2B). Furthermore, we observed a dose-dependent increase in the expression of the phospho(Y)-PITX1 after PTP1B inhibitor treatment (Fig. 4C, left panel). Moreover, in line with the change in phospho(Y)-PITX1, the expression of p120RasGAP and PITX1 was also upreguated by PTP1B inhibitor treatment (Fig. 4C, right panel, Supplement Figure 3A,B). Collectively, the above data indicated that PTP1B dephosphorylates PITX1 at certain tyrosine residue(s) that results in destabilization of PITX1 and downregulation of p120RasGAP.

### PTP1B dephosphorylates PITX-1 at Y160, 175 and Y179

To clarify which tyrosine residue(s) are involved in PTP1B-mediated PITX1 dephosphorylation, we applied NetPhos 2.0 Server^24^, and three potential tyrosine residues, Y160, Y175 and Y179, were identified. We generated three site-specific mutations of PITX1, namely Y160F, Y175F and Y179F, and examined the effects of PIP1B on DLD1 cells with transient expression of these mutation constructs. As shown in Fig. 4D, distinct from that observed in cells expressing wild-type PITX1, the phospho(Y)-status of all three mutations were not affected by PTP1B overexpression, indicating that PTP1B potentially dephosphorylates PITX-1 at these three tyrosine residues. Furthermore, we exposed DLD1 cells expressing the wild-type and the three mutant forms of PITX-1 to cycloheximide (CHX) treatment, a protein synthesis inhibitor. Interestingly, we found that degradation of PITX-1 protein was significantly enhanced in the above-mentioned mutations in comparison with the wild-type PITX1 (Fig. 4E and Supplement Figure 3C), implying that these three tyrosine sites are critical in determining the stability of PITX-1 protein. Moreover, Lactacystin, a proteasome inhibitor, induced potent augmentation of PITX-1 expression in cells expressing wild-type PITX1; but such effects were lost in cells expressing mutant PITX1 (Fig. 4F). Collectively, our data suggest that PTP1B has a critical role in promoting the proteasomal degradation of PITX1 through dephosphorylating PITX-1 at Y160, Y175 and Y179.

### Regorafenib inhibits the phosphatase activity of PTP1B and upregulates the expression of PITX-1/p120RasGAP

**Figure 5:**
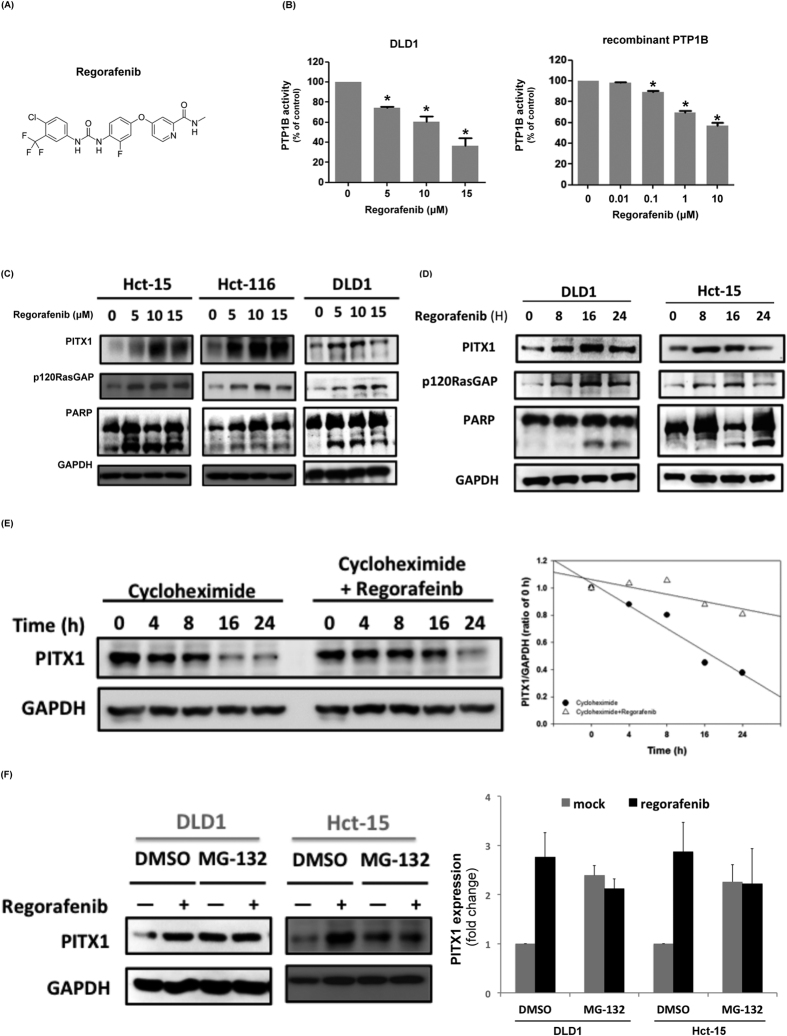
Regorafenib upregulates PITX1/p120RasGAP axis through inactivation of PTP1B. (**A**) Chemical structure of regorafenib. (**B**) Effects of regorafenib on PTP1B phosphatase activities were assessed in DLD1 cells (left panel) and recombinant PTP1B (right panel) after exposure to regorafenib at the indicated doses. Treatment duration for DLD1 cell was 16 h and 4 h for recombinant PTP1B protein. (**C**) Regorafenib dose-dependently induced upregulation of PITX-1 and RasGAP and promoted apoptosis in CRC cells. N = 3. Representative image of western blot were shown here. (**D**) Time-dependent effects of regorafenib on PITX1/p120RasGAP axis and apoptosis related signaling in CRC cells. N = 3. Representative image of western blot were shown here. (**E**) Regorafenib treatment delayed the turnover of PITX1 protein. After exposure to 50 μg/ml cycloheximide (CHX) alone or co-treatment with regorafenib 5 μM at the indicated time, PITX1 protein expression in DLD1 cell were examined by western blot. N = 3. Representative images of western blot were shown over the left side, and the quantified result of PITX1 expression was shown over the right panel. N = 3. (**F**) Regorafenib-induced augmentation of PITX1 expression was diminished in MG-231-treated cells. Effects of regorafenib on PITX1 protein expression in DLD1 and Hct-15 cells were determined after pretreated to MG-231, the proteasome inhibitor, or DMSO. Quantified results of the PITX1 expression in each treatment obtained in the triplicated experiments were shown over the right panel.

Given the known crucial role of RAS signaling in CRC and the novel mechanism of PTP1B in regulating p120RasGAP that we have characterized in the current project, we were interested to know whether targeting PTP1B exerts therapeutic potential against CRC. Thus, we explored the role of PTP1B/PITX1/p120RasGAP signaling as associated with the effects of regorafenib (Fig. 5A), an approved oral target agent for the treatment of CRC. Interestingly, we found that regorafenib inhibited the phosphatase activity of PTP1B. As shown in Fig. 5B, regorafenib dose-dependently decreased the PTP1B phosphatase activity in DLD1 cell lysate (left panel) and recombinant PTP1B protein (right panel). Furthermore, we found that the expressions of PITX1 and p120RasGAP, which are associated with activation of apoptotic signals, were correspondingly upregulated by regorafenib treatment in a dose- and time-dependent manner (Fig. 5C,D and Supplement Figure 4A,B). Previously, we showed that PTP1B affects the expression of PITX-1/RasGAP by promoting proteasomal degradation of PITX-1 (Fig. 4E,F). Thus, we checked whether the effects of regorafenib on PITX1 expression did act on preventing its degradation. We examined the half-life of PITX1 by exposing DLD1 cells to CHX and/or regorafenib. As shown in Fig. 5E and Supplement Figure 4C, regorafenib indeed delayed the protein turnover of PITX1. Moreover, we used MG-132, a specific proteasome inhibitor, to validate the role of proteasome degradation in mediating the effects of regorafenib. In line with our hypothesis, we showed that regorafenib-induced PITX1 augmentation was blocked by MG-132 in DLD1 and Hct-15 cells (Fig. 5F and Supplement Figure 4D). Collectively, our data suggested that regorafenib potentially affects Ras activity by inhibiting PTP1B-dependent PITX1-p120RasGAP downregulation.

### Direct interaction with the PTP1B/PITX1/p120RasGAP axis determines the anti-CRC effects of regorafenib

**Figure 6:**
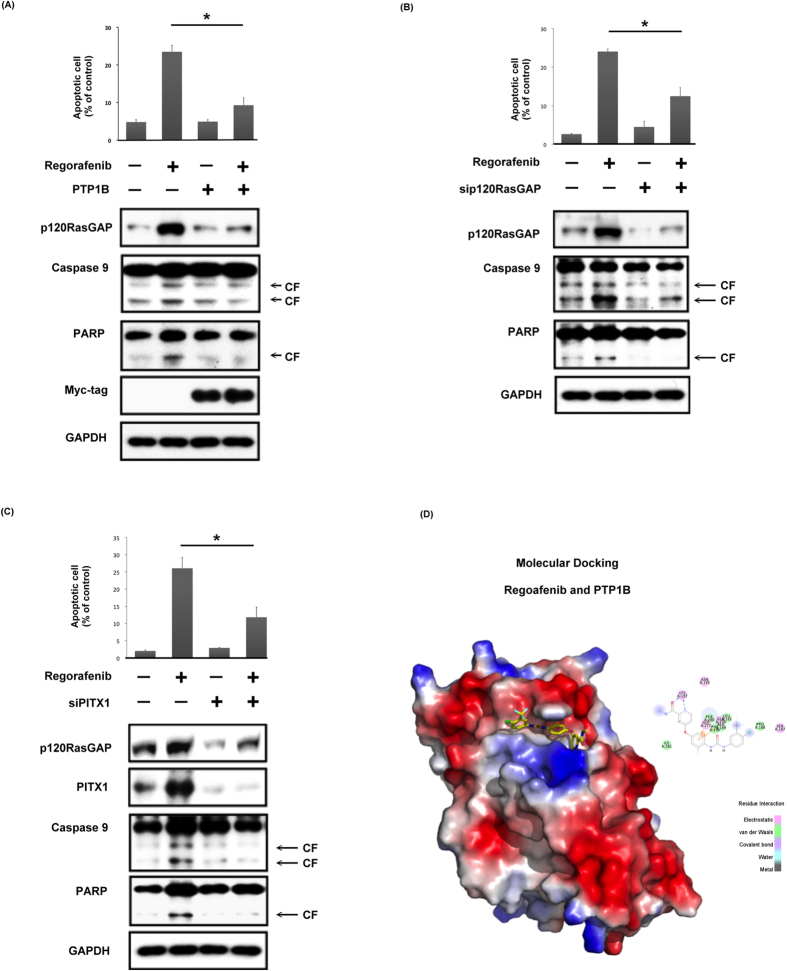
Validation of the role of PTP1B/PITX1/p120RasGAP signaling in determining the anti-CRC effects of regorafenib. (**A**) Overexpression of PTP1B diminished the effects of regorafenib on affecting p120RasGAP expression and promotion of apoptosis in CRC cells. DLD1 cells transiently expressing PTP1B with Myc-tag were exposed to 5 μM regorafenib for 24 hours. The percentage of apoptosis was determined by sub-G1 analysis and alterations of molecular signaling were determined by western blot. Columns, mean; bars, SD (*n* = 3); **P < 0.05. N = 3. (**B**) Silencing of p120RasGAP reduced the extent of regorafenib-induced apoptosis of CRC cells. The percentage of apoptosis was determined by sub-G1 analysis. Columns, mean; bars, SD (*n* = 3); **P < 0.05. (**C**) Knockdown of PITX1 diminished the effects of regorafenib-induced upregulation of p120RasGAP expression and apoptosis of CRC cells. The percentage of apoptosis was determined by sub-G1 analysis and alterations of molecular signaling were determined by western blot. Columns, mean; bars, SD; *P < 0.05. N = 3. (**D**) Modeled docking of the interaction between regorafenib and PTP1B protein (PDB ID: 1T49). The potential docking site of regorafenib into the catalytic site of PTP1B protein was shown by the riboon diagram. The GOLD fitness score of regorafenib and PTP1B was 78.61.

To validate our findings on the novel mechanism of regorafenib, we further manipulated the level of PTP1B, PITX1 and p120RasGAP and examined their impacts on affecting the effectiveness of regorafenib. First, we overexpressed PTP1B in DLD1 cells and examined the effects of regorafenib. As shown in Fig. 6A, regorafenib treatment led to significant upregulation of RasGAP and apoptosis of DLD1 cells, and these effects were diminished by overexpression of PTP1B. On the other hand, DLD1 cells with silencing of p120RasGAP (*RASAL1,* Fig. 6B) and PITX1 (*PITX1,* Fig. 6C) reversed the effects of regorafenib on promoting apoptosis. Furthermore, we performed molecular docking to dissect the potential docking sites of regorafenib in PTP1B. As shown in Fig. 6D, regorafenib was docked into a groove between helices α3 and α6 in the catalytic domain of PTP1B, and interacted with the WPD loop via electrostatic and von der Waals force. Collectively, our data illustrates a new mechanism of action of regorafenib in CRC, i.e. direct targeting the novel oncoprotein, PTP1B, and subsequent upreguation of the expression of PITX-1/RasGAP.

### Inhibition of PTP1B mediates the *in-vivo* anti-tumor effects of regorafenib

**Figure 7:**
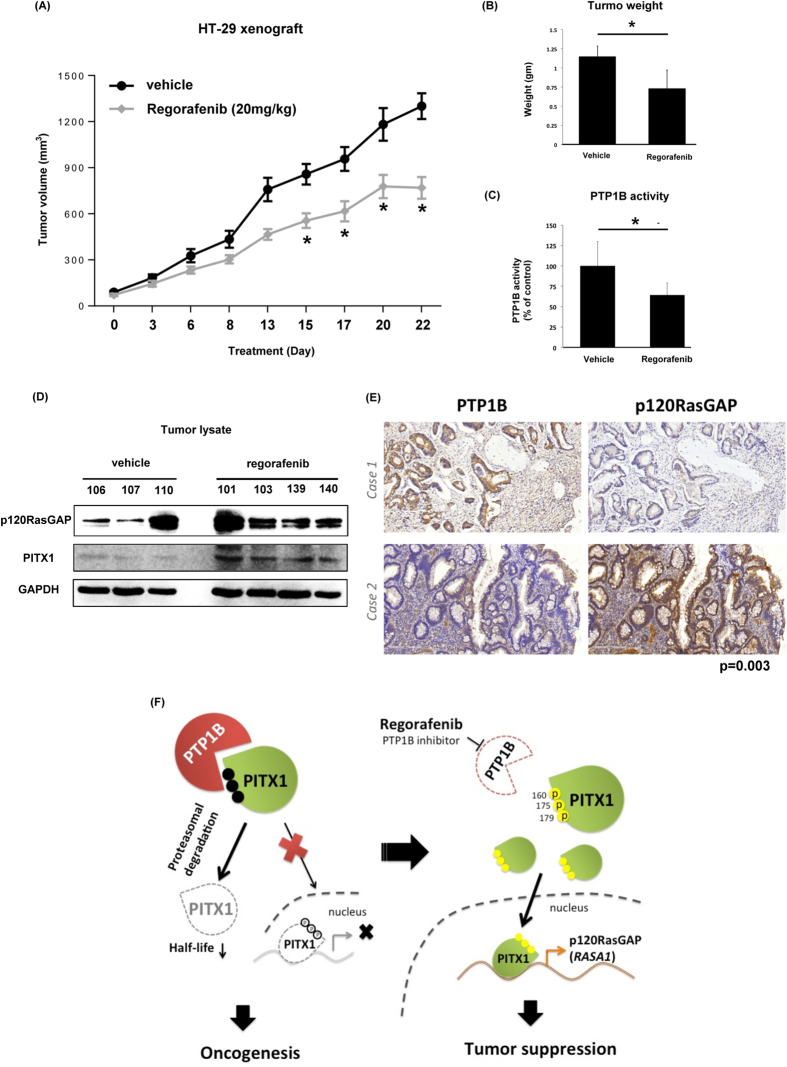
*In vivo* effects of regorafenib on HT-29 subcutaneous xenograft tumor model. (**A**) The growth curve of HT-29 xenograft tumor in nude mice exposing to regorafenib or vehicle control. (N = 10 in each group) *P < 0.05. (**B**) Average weights of HT-29 xenograft tumors obtained at the end of treatment. Columns, mean; bars, SD; *P < 0.05. (**C**) PTP1B activities of regorafenib- and vehicle-treated HT-29 xenograft tumors. Columns, mean; bars, SD; *P < 0.05. (**D**) The expressions of PITX1 and p120RasGAP in the regorafenib- and vehicle-treated HT-29 xenograft tumors. (**E**) The expressions of PTP1B and RasGAP in clinical CRC tumors were analyzed in the CRC cohort containing 242 patients, and the correlation between these proteins were analyzed by Chi-square. P = 0.003. (**F**) Summary model. Regorafenib enhanced the expression of PITX1/p120RasGAP through inhibiting PTP1B-promoted PITX1 degradation.

To validate the *in vivo* effects of regorafenib on the PTP1B-PITX-1-RasGAP axis, we tested the effects of regorafenib in a clinically relevant CRC animal model. As shown in Fig. 7A, regorafenib treatment significantly inhibited the growth rate of HT29 subcutaneous xenograft tumor. Average tumor weight was also reduced in the regorafenib arm (Fig. 7B). Importantly, the PTP1B activity was significantly inhibited in the tumor lysate obtained from regorafenib-treated mice (Fig. 7C). In agreement, the expression of PITX-1 and RasGAP in mice treated with regorafenib was higher than mock-treated mice (Fig. 7D). Furthermore, we validated the clinical relevance our finding by examining the clinical tumor samples from the cohort we characterized above. First, we found a negative association between PTP1B and p120RasGAP expression (*P* = 0.003, Fig. 7E). More interestingly, we showed that the expression level of PTP1B was significantly correlated with p-ERK, one important downstream factor of Ras signaling (*P* = 0.001, Supplement Figure 5B). Moreover, CRC patient with low- or non-expression of PTP1B and p-ERK had superior overall survival than the others (*P* = 0.05). Concluded from the findings in CRC animal model and the clinical tumors consolidate the role of PTP1B in regulating Ras signaling and mediating the therapeutic effects of regorafenib. Further study to investigate the potential of targeting PTP1B for the treatment of CRC is warranted (Fig. 7F).

## Discussion

Given the complex network of signaling that PTP1B involved, the “net effect” of PTP1B plays in individual cancer may be different and should be discussed separately. In the current work, we showed that PTP1B is sufficient and necessary for the maintenance of CRC cells (Figs 2 and 5 and supplement Figure 1). Furthermore, we, for the first time, described how this novel oncoprotein interacts with the Ras signaling in CRC. Ras signaling has long been known as one of the fundamental mechanisms that drives colon carcinogenesis^3^. Occurrence of the activating mutations of Ras gene in codon 12, 13 and 61 is regarded as the most common cause explaining aberrant activation of Ras signaling in cancer. However, only half of CRC patients presented with detectable genetic mutation of the *Ras* gene^21^. Thus, it is of great interest to explore other regulatory mechanisms of Ras signaling associated with the development of CRC. Here, we showed that PTP1B downregulated the expression of p120RasGAP via destabilizing PITX-1, the transcription factor of p120RasGAP in CRC cells (Figs 3 and 4). Through directly dephosphorylating PITX-1 at Y160, Y175 and Y179, PTP1B promoted proteasomal degradation of PITX-1, thus leaded in downregulating p120RasGAP and CRC cell survival. Above findings were not only validated vigorously be a serial knockdown and overexpression experiments, but also echoed the findings we observed in hepatocellular carcinoma cells (HCC)^25^. Given the distinct biological backgrounds, especially regarding RAS mutation status, the commonality of PTP1B we found in CRC and HCC is interesting, and further studies is warranted.

The activity of Ras protein is controlled by the ratio of bound GTP to GDP, and alternation between the active Ras-GTP and inactive Ras-GDP is regulated by guanine nucleotide exchange factors, which stimulate the exchange of GDP for GTP, and GAPs, which terminate the active state by stimulating GTP hydrolysis^26^,^27^. Interestingly, accumulating evidence suggests that deregulation of GAPs has a critical role in promoting carcinogenesis. For example, germline mutational loss of neurofibromin (NF1), a member of the Ras GAP family, resulted in the autosomal dominantly inherited disorder neurofibromatosis type 1, which is known to increase risk of developing neuroblastoma or other tumors of the peripheral and central nervous system^28^,^29^. p120RasGAP is a member of the Ras GAPs^30^, and its roles in colon cancer has been discussed in the work presented by Ohta *et al*., which showed that decreased expression of p120RasGAP by epigenetic silencing was associated with colorectal tumor progression^31^. Ectopic overexpression of p120RasGAP defected the growth rate of CRC cells, while knockdown of p120RasGAP significantly potentiated the growth rate of PMFko14 xenograft tumor^31^. For other cancer types, somatic inactivation of p120RasGAP was reported in thyroid, nasopharyngeal, esophageal and gastric cancer^32^,^33^,^34^, and a potential tumor suppressor role of p120RasGAP in basal cell carcinoma, and triple negative and HER2 positive subtypes of breast cancer was also suggested by genetic studies and integrative bioinformatics analysis^35^,^36^. Notably, Ingrid *et al*. demonstrated the effects of PITX1 on transcriptionally upregulating the expression of *RASAL*1 that resulted in suppression of Ras activity and tumorigenicity of colon cancer cells^22^. Furthermore, low levels of expression of PITX1 were found to be predict worse survival of CRC patients^37^. Together with our data, dysregulation of PITX-1/p120RasGAP axis may be an important mechanism, in addition to mutation of Ras gene, explaining deregulated Ras signaling in cancer cells.

In the present study, we reported for the first time that regorafenib inhibits PTP1B activity in CRC (Figs 5, 6 and 7). Treatment with regorafenib significantly delayed the turnover of PITX-1 protein and upregulated the expression of PITX-1 and p120RasGAP in a dose- and time-dependent manner through inactivating PTP1B (Fig. 5). Importantly, the effects of regorafenib on the PITX-1/RasGap axis and induction of cell apoptosis were significantly diminished in cells with overexpression of PTP1B and knockdown of PITX1 or p120RasGAP (Fig. 6). Importantly, our results suggest that regorafenib inhibited the activity of PTP1B by direct interaction with its catalytic domain (Fig. 4B, right panel, and Fig. 6D). The idea of using PTP1B inhibitor as an anti-cancer treatment was first suggested by observing that a specific PTP1B inhibitor significantly delayed the development of breast tumor in NDL2 transgenic mice via attenuating the MAPK and Akt pathways^38^. Recently, Krishnan *et al*. also showed the efficacy of MSI-1436, a novel allosteric inhibitor of PTP1B, against HER2-driven breast tumor and abrogated the development of pulmonary metastasis^39^. Our data suggest the exciting possibility of applying PTP1B inhibitor against CRC. Given the well-known roles of PTP1B in regulating insulin and leptin signaling, the development of a PTP1B inhibitor for the treatment of diabetes and obesity is already in progress^10^,^11^,^40^,^41^,^42^. Therefore, it is worth further exploring the effectiveness of these potent and selective PTP1B inhibitors for the treatment of cancer, especially the cancer types like CRC and breast cancer that are associated with metabolism. In addition, our data indicated that PTP1B expression may be a novel biomarker to predict effectiveness of regorafenib treatment in CRC patients. Further studies are required to explore this issue.

As described before, PTP1B is an important regulator of cell metabolism and has a strong link with the pathogenesis of metabolic syndrome and diabetes. In this project, in addition to dissect the association of PTP1B and the clinical aggressiveness of CRC patients, we also tried to known whether PTP1B may link metabolic disorder to colon carcinogenesis. By analyzing clinical tumor samples with immunohistochemical stain, we showed that overexpression of PTP1B is a recurrent event in CRC patients and associated with a shorter metastatic-free survival and overall survival (Fig. 1). Our results echoed the findings of the other two groups^18^,^19^. Furthermore, we showed a significant positive correlation between PTP1B and p-Erk, one important indicator for activation of Ras signaling (Supplement Figure 5), supporting the importance of PTP1B in regulating Ras signaling. Interestingly, we also found a trend of increasing risk in CRC patients with PTP1B overexpression to be co-morbid with type 2 diabetes mellitus (data not shown). Above findings not only add knowledge to the oncogenic properties of PTP1B in CRC, but also offer new clues to link insulin resistance with colon carcinogenesis.

In this study, we reported a novel oncogenic property of PTP1B in CRC. By direct dephosphorylation of PITX1 at three potential tyrosine residues, PTP1B promotes proteasome degradation of PITX1 and downregulation of p120RasGAP. By directly inhibiting the phosphatase function of PTP1B, regorafenib restored the expression of PITX1/p120RasGAP and promoted apoptosis of CRC cells.

## Material and Methods

### Cell culture, reagents and antibodies

The colorectal cancer (CRC) cell lines Hct-15, Hct-116, SW-480, DLD1 and HT-29 were maintained in RPMI 1640 medium with 10% fetal bovine serum (FBS); and CCD-18Co, the normal colon fibroblast cell line, was maintained in Eagle’s Minimum Essential medium supplemented with 10% FBS. All cells were incubated at 37 °C in a humidified 5% CO_2_ atmosphere. Regorafenib (BAY 73-4506) was purchased from Selleck Chemicals and PTP1B inhibitor was purchased from Calbiochem. Lactacystin and MG-132 were obtained from Sigma. For *in vitro* studies, drugs were dissolved in dimethyl sufoxide (DMSO) at various concentrations and added to cells in RPMI 1640 medium. The final DMSO concentration was 0.1% after adding to the medium. Antibodies for immunoblotting including Caspase-9 and Myc-tag were purchased from Cell Signaling (Danvers, MA); anti- PARP-1 and anti-PTP1B were from Santa Cruz Biotechnology (San Diego, CA); anti-p-Try was obtained from Millipore (Billerica, MA). Others including anti-PITX-1, -RASA1 (RasGAP) and anti-GAPDH were all obtained from Abcam (Cambridge, MA).

### Cell proliferation, colony formation and sphere formation assay

The MTT assay was used to determine the proliferation of CRC cells with/without ectopic expression of PTP1B. In brief, 2000 of the indicated cells were seeded at each well of a 96-well plate and maintained in 10% FBS culture medium. After 72 hours, 1 mg/ml MTT was added to each well, incubated for three hours, and analyzed by ELISA reader at 590 nm. For colony formation assay, DLD1 cells with and without ectopic expression of PTP1B and Hct-116 cells with and without PTP1B knockdown were seeded in 10 cm plates at a density of 500 cells per well and culture for 14 days. Colonies were fixed with 4% of paraformaldehyde solution, stained with 0.5% crystal violet, and analyzed by inverted microscope. For the generation of spheres, 500 DLD1 cells with PTP1B ectopic expression or PTP1B knockdown were plated in 24-well ultra-low attachment plates (Corning) and grown in median containing 10% FBS or serum free condition as indicated in each experiment. After indicated time, the number of tumor sphere was calculated and analyzed.

### Apoptosis assay and western blot analysis

The extent of apoptosis induced by indicated treatments was determined by flow cytometry (sub-G1) and western blot analysis of PARP and Caspase 9. Sub-G1 analysis, western blot analysis and co-immunoprecipitation were performed as previously described^43^. Image J was used to quantify protein expression.

### Plasmids, siRNA and transfection

The potential phosphorylation sites of PITX1 affected by PTP1B was predicted by the NetPhos 2.0 Server (http://www.cbs.dtu.dk/services/NetPhos/)^24^, and we generated three Y/F mutant constructs of PTP1B, namely Y160, Y175, and Y179. Subsequently, we confirmed the DNA sequence and expression level in CRC cells. For subsequent transfection experiments, we used pCMV6-Entry vector with myc-tag to generate the plasmids encoding the human PTP1B, PITX1 and PTP1B mutants. For siRAN experiments, we used smart-pool siRNA from Dharmacon (Chicago, IL), including control (D-001810-10), PTP1B (PTPN1, L-003529-00-0005), RASA1 (L-005276-00-0005), and PITX1 (L-017246-00-0005). All the experiments were carried out in a transient expression fashion. Plasmids or siRNA were first incubated with lipofectamine 2000 (Invitrogen, CA) or DharmaFECT4 (Dharmacon, Chicago, IL) for 24 h and then processed with the indicated treatment for another 24 h.

### PTP1B phosphatase activity

For *in-vivo* PTP1B phosphatase activity assessment, lysate of regorafenib-treated DLD1 cells was first incubated overnight with buffer containing with anti-PTP1B antibody for co-immunoprecipitation, In the next day, we added protein G-Sepharose 4 Fast Flow (GE Healthcare Bio-Science, NJ) into each sample and incubated for another 3 hours. The samples were kept at 4 °C condition with rotation. For *ex-vivo* assessment, recombinant PTP1B proteins (Genway, GWB-IE1774) was first treated with regorafenib for 4 hours and assessed by a commercial kit, RediPlate 96 EnzChek Tyrosine Phosphatase Assay Kit (R-22067) for PTP1B activity assay (Molecular Probes, Invitrogen, CA).

### Animal study

For *in vivo* testing, we used male NCr athymic nude mice (5–7 weeks of age) obtained from the National Laboratory Animal Center (Taipei, Taiwan). All the experimental design and protocol of this study were reviewed and approved by the Institutional Laboratory Animal Care and Use Committee of National Taiwan Universit, and all experiments were conducted strictly in accordance with protocols approved.

In brief, we first inoculated a subcutaneous tumor with 2 × 10^6^ HT29 cells suspended in 0.1 ml of serum-free medium containing 50% Matrigel (BD Biosciences, Bedford, MA) over the dorsal flank of mouse. Treatment with regorafenib (10 mg/kg) or vehicle was started when the tumor reached 100–200 mm^3^. All treatment was given orally at everyday fashion. To evaluate the efficacy of each treatment, we measured the size of these subcutaneous tumors twice a week. The volume of the tumor was approximated by the following formula: width × length × height × 0.523. At the end of experiment, we harvested the tumor samples for western blot and PTP1B activity assay.

### Immunohistochemistry

The study protocol and all the human experiment were approved and conduced according to the guidance of the ethics committee of the Institutional Review Board of Taipei Veterans’ General Hospital. For every patient enrolled in this study, a informed consent, accordance with the Declaration of Helsinki, were obtained at the time of donation. Before performing IHC staining, we took the representative CRC tumors and the adjacent normal tissues from each patients and made into tissue array. For IHC experiment, the slides containing paraffin-embedded CRC tissue sections were first deparaffinized and rinsed with 10 mmol/L Tris-HCl (pH 7.4) and 150 mmol/L sodium chloride, followed by methanol and 3% hydrogen peroxide to quench the effects of peroxidase. Before staining, slides were heated in citrate buffer (10 mmol/L, pH 6.0) at 100 °C for 20 minutes for antigen retrieval. Afterward, slides were exposed to primary antibodies, namely, PTP1B, p120RasGAP, and p-Erk, for one hour at room temperature. After throughout wash with PBS, we used EnVision Detection Systems Peroxidase/DAB, Rabbit/Mouse Kit (Dako) to detect the signal. For all the experiments, we used prior-confirmed positive samples as positive control and had primary antibody replaced by PBS as negative control. All the slides were reviewed by a board certified pathologist for the intensity and the percentage of stained cells.

### Statistical Analysis

We used independent samples *t* test to compare the means between each tests and applied linear regression analysis to investigate the correlation of the expression of PITX-1 and RasGAP. Overall survival were defined as the time of CRC diagnosis to the confirmed date of mortality or last confirmed follow-up in all cohort and metastatic-free survival were calculated from the date of CRC diagnosis to the date of confirmed metastatic lesion caused by CRC or last follow-up in patients with stage I-III diseases at diagnosis. Kaplan-Meier method was used to estimate the survival and the log-rank test was used for compare between defined groups. All the statistical analyses conducted by SPSS software for Windows (SPSS, Chicago, 17.0 version, IL, USA).

## Additional Information

**How to cite this article**: Teng, H.-W. *et al*. Protein tyrosine phosphatase 1B targets PITX1/p120RasGAP thus showing therapeutic potential in colorectal carcinoma. *Sci. Rep.* **6**, 35308; doi: 10.1038/srep35308 (2016).

## Supplementary Material

Supplementary Information

## References

[b1] Torre L. A. . Global cancer statistics, 2012. CA: a cancer journal for clinicians 65, 87–108, 10.3322/caac.21262 (2015). 25651787

[b2] Haggar F. A. & Boushey R. P. Colorectal cancer epidemiology: incidence, mortality, survival, and risk factors. Clinics in colon and rectal surgery 22, 191–197, 10.1055/s-0029-1242458 (2009). 21037809 PMC2796096

[b3] Fearon E. R. & Vogelstein B. A genetic model for colorectal tumorigenesis. Cell 61, 759–767 (1990). 2188735 10.1016/0092-8674(90)90186-i

[b4] Giovannucci E. . Physical activity, obesity, and risk for colon cancer and adenoma in men. Annals of internal medicine 122, 327–334 (1995). 7847643 10.7326/0003-4819-122-5-199503010-00002

[b5] Inoue M. & Tsugane S. Insulin resistance and cancer: epidemiological evidence. Endocrine-related cancer 19, F1–F8, 10.1530/erc-12-0142 (2012). 22851686

[b6] Campbell P. T. . Prospective study reveals associations between colorectal cancer and type 2 diabetes mellitus or insulin use in men. Gastroenterology 139, 1138–1146, 10.1053/j.gastro.2010.06.072 (2010). 20633560

[b7] Ostman A. & Bohmer F. D. Regulation of receptor tyrosine kinase signaling by protein tyrosine phosphatases. Trends in cell biology 11, 258–266 (2001). 11356362 10.1016/s0962-8924(01)01990-0

[b8] Dube N., Cheng A. & Tremblay M. L. The role of protein tyrosine phosphatase 1B in Ras signaling. Proceedings of the National Academy of Sciences of the United States of America 101, 1834–1839, 10.1073/pnas.0304242101 (2004). 14766979 PMC357013

[b9] Saltiel A. R. & Kahn C. R. Insulin signalling and the regulation of glucose and lipid metabolism. Nature 414, 799–806, 10.1038/414799a (2001). 11742412

[b10] Seely B. L. . Protein tyrosine phosphatase 1B interacts with the activated insulin receptor. Diabetes 45, 1379–1385 (1996). 8826975 10.2337/diab.45.10.1379

[b11] Zabolotny J. M. . PTP1B regulates leptin signal transduction *in vivo*. Developmental cell 2, 489–495 (2002). 11970898 10.1016/s1534-5807(02)00148-x

[b12] Cheng A. . Attenuation of leptin action and regulation of obesity by protein tyrosine phosphatase 1B. Developmental cell 2, 497–503 (2002). 11970899 10.1016/s1534-5807(02)00149-1

[b13] LaMontagne K. R. Jr., Hannon G. & Tonks N. K. Protein tyrosine phosphatase PTP1B suppresses p210 bcr-abl-induced transformation of rat-1 fibroblasts and promotes differentiation of K562 cells. Proceedings of the National Academy of Sciences of the United States of America 95, 14094–14099 (1998). 9826659 10.1073/pnas.95.24.14094 PMC24332

[b14] Balsamo J. . Regulated binding of PTP1B-like phosphatase to N-cadherin: control of cadherin-mediated adhesion by dephosphorylation of beta-catenin. The Journal of cell biology 134, 801–813 (1996). 8707857 10.1083/jcb.134.3.801 PMC2120944

[b15] Eden E. R., White I. J., Tsapara A. & Futter C. E. Membrane contacts between endosomes and ER provide sites for PTP1B-epidermal growth factor receptor interaction. Nature cell biology 12, 267–272, 10.1038/ncb2026 (2010). 20118922

[b16] Yip S. C., Saha S. & Chernoff J. PTP1B: a double agent in metabolism and oncogenesis. Trends in biochemical sciences 35, 442–449, 10.1016/j.tibs.2010.03.004 (2010). 20381358 PMC2917533

[b17] Zhu S., Bjorge J. D. & Fujita D. J. PTP1B contributes to the oncogenic properties of colon cancer cells through Src activation. Cancer research 67, 10129–10137, 10.1158/0008-5472.can-06-4338 (2007). 17974954

[b18] Hoekstra E. . Increased PTP1B expression and phosphatase activity in colorectal cancer results in a more invasive phenotype and worse patient outcome. Oncotarget, 10.18632/oncotarget.7829 (2016). PMC5008334 26942883

[b19] Chen Q., Li Y., Li Z., Zhao Q. & Fan L. Overexpression of PTP1B in human colorectal cancer and its association with tumor progression and prognosis. Journal of molecular histology 45, 153–159, 10.1007/s10735-013-9536-1 (2014). 23990346

[b20] Mor A., Aizman E., George J. & Kloog Y. Ras inhibition induces insulin sensitivity and glucose uptake. PloS one 6, e21712, 10.1371/journal.pone.0021712 (2011). 21738773 PMC3126849

[b21] Pylayeva-Gupta Y., Grabocka E. & Bar-Sagi D. RAS oncogenes: weaving a tumorigenic web. Nature reviews. Cancer 11, 761–774, 10.1038/nrc3106 (2011). 21993244 PMC3632399

[b22] Kolfschoten I. G. . A genetic screen identifies PITX1 as a suppressor of RAS activity and tumorigenicity. Cell 121, 849–858, 10.1016/j.cell.2005.04.017 (2005). 15960973

[b23] Grothey A. . Regorafenib monotherapy for previously treated metastatic colorectal cancer (CORRECT): an international, multicentre, randomised, placebo-controlled, phase 3 trial. Lancet (London, England) 381, 303–312, 10.1016/s0140-6736(12)61900-x (2013). 23177514

[b24] Blom N., Gammeltoft S. & Brunak S. Sequence and structure-based prediction of eukaryotic protein phosphorylation sites. Journal of molecular biology 294, 1351–1362, 10.1006/jmbi.1999.3310 (1999). 10600390

[b25] Tai W. T. . Protein tyrosine phosphatase 1B dephosphorylates PITX1 and regulates p120RasGAP in hepatocellular carcinoma. Hepatology (Baltimore, Md.) 63, 1528–1543, 10.1002/hep.28478 (2016). 26840794

[b26] Vigil D., Cherfils J., Rossman K. L. & Der C. J. Ras superfamily GEFs and GAPs: validated and tractable targets for cancer therapy? Nature reviews. Cancer 10, 842–857, 10.1038/nrc2960 (2010). 21102635 PMC3124093

[b27] Grewal T., Koese M., Tebar F. & Enrich C. Differential Regulation of RasGAPs in Cancer. Genes & cancer 2, 288–297, 10.1177/1947601911407330 (2011). 21779499 PMC3128632

[b28] Brems H., Beert E., de Ravel T. & Legius E. Mechanisms in the pathogenesis of malignant tumours in neurofibromatosis type 1. The Lancet. Oncology 10, 508–515, 10.1016/s1470-2045(09)70033-6 (2009). 19410195

[b29] Laycock-van Spyk S., Thomas N., Cooper D. N. & Upadhyaya M. Neurofibromatosis type 1-associated tumours: their somatic mutational spectrum and pathogenesis. Human genomics 5, 623–690 (2011). 22155606 10.1186/1479-7364-5-6-623 PMC3525246

[b30] Maertens O. & Cichowski K. An expanding role for RAS GTPase activating proteins (RAS GAPs) in cancer. Advances in biological regulation 55, 1–14, 10.1016/j.jbior.2014.04.002 (2014). 24814062

[b31] Ohta M. . Decreased expression of the RAS-GTPase activating protein RASAL1 is associated with colorectal tumor progression. Gastroenterology 136, 206–216, 10.1053/j.gastro.2008.09.063 (2009). 18992247

[b32] Jin H. . Epigenetic silencing of a Ca(2+)-regulated Ras GTPase-activating protein RASAL defines a new mechanism of Ras activation in human cancers. Proceedings of the National Academy of Sciences of the United States of America 104, 12353–12358, 10.1073/pnas.0700153104 (2007). 17640920 PMC1941473

[b33] Liu D., Yang C., Bojdani E., Murugan A. K. & Xing M. Identification of RASAL1 as a major tumor suppressor gene in thyroid cancer. Journal of the National Cancer Institute 105, 1617–1627, 10.1093/jnci/djt249 (2013). 24136889 PMC3818169

[b34] Seto M. . Reduced expression of RAS protein activator like-1 in gastric cancer. International journal of cancer. Journal international du cancer 128, 1293–1302, 10.1002/ijc.25459 (2011). 20473946

[b35] Hu X. . Genetic alterations and oncogenic pathways associated with breast cancer subtypes. Molecular cancer research: MCR 7, 511–522, 10.1158/1541-7786.mcr-08-0107 (2009). 19372580

[b36] Friedman E., Gejman P. V., Martin G. A. & McCormick F. Nonsense mutations in the C-terminal SH2 region of the GTPase activating protein (GAP) gene in human tumours. Nature genetics 5, 242–247, 10.1038/ng1193-242 (1993). 8275088

[b37] Knosel T. . Loss of desmocollin 1-3 and homeobox genes PITX1 and CDX2 are associated with tumor progression and survival in colorectal carcinoma. International journal of colorectal disease 27, 1391–1399, 10.1007/s00384-012-1460-4 (2012). 22438068

[b38] Julien S. G. . Protein tyrosine phosphatase 1B deficiency or inhibition delays ErbB2-induced mammary tumorigenesis and protects from lung metastasis. Nature genetics 39, 338–346, 10.1038/ng1963 (2007). 17259984

[b39] Krishnan N. . Targeting the disordered C terminus of PTP1B with an allosteric inhibitor. Nature chemical biology 10, 558–566, 10.1038/nchembio.1528 (2014). 24845231 PMC4062594

[b40] Thareja S., Aggarwal S., Bhardwaj T. R. & Kumar M. Protein tyrosine phosphatase 1B inhibitors: a molecular level legitimate approach for the management of diabetes mellitus. Medicinal research reviews 32, 459–517, 10.1002/med.20219 (2012). 20814956

[b41] Popov D. Novel protein tyrosine phosphatase 1B inhibitors: interaction requirements for improved intracellular efficacy in type 2 diabetes mellitus and obesity control. Biochemical and biophysical research communications 410, 377–381, 10.1016/j.bbrc.2011.06.009 (2011). 21683066

[b42] Combs A. P. Recent advances in the discovery of competitive protein tyrosine phosphatase 1B inhibitors for the treatment of diabetes, obesity, and cancer. Journal of medicinal chemistry 53, 2333–2344, 10.1021/jm901090b (2010). 20000419

[b43] Tai W. T. . Discovery of novel Src homology region 2 domain-containing phosphatase 1 agonists from sorafenib for the treatment of hepatocellular carcinoma. Hepatology (Baltimore, Md.) 59, 190–201, 10.1002/hep.26640 (2014). 23908138

