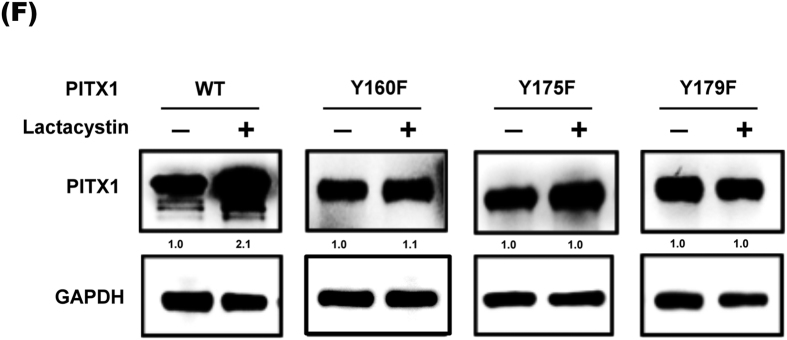# Corrigendum: Protein tyrosine phosphatase 1B targets PITX1/p120RasGAP thus showing therapeutic potential in colorectal carcinoma

**DOI:** 10.1038/srep45612

**Published:** 2017-04-10

**Authors:** Hao-Wei Teng, Man-Hsin Hung, Li-Ju Chen, Mao-Ju Chang, Feng-Shu Hsieh, Ming-Hsien Tsai, Jui-Wen Huang, Chih-Lung Lin, Hsiang-Wen Tseng, Zong-Keng Kuo, Jeng-Kai Jiang, Shung-Haur Yang, Chung-Wai Shiau, Kuen-Feng Chen

Scientific Reports 6: Article number: 3530810.1038/srep35308; published online: 10 18 2016; updated: 04 10 2017

This Article contains an error in Figure 4F of this Article. The immunoblot for GAPDH is incorrect. The correct Figure 4F appears below as Figure 1.

## Figures and Tables

**Figure 1:**