# Supplementary material for: Protein tyrosine phosphatase 1B targets PITX1/p120RasGAP thus showing therapeutic potential in colorectal carcinoma
Source: Sci Rep. 2016 Oct 18;6:35308. doi: 10.1038/srep35308 (PMC5082755; doi:10.1038/srep35308)
Supplement: Supplementary Information [file srep35308-s1.pdf]

Protein tyrosine phosphatase 1B targets PITX1/p120RasGAP thus showing therapeutic potential in colorectal carcinoma

Hao-Wei Teng, Man-Hsin Hung, Li-Ju Chen, Mao-Ju Chang, Feng-Shu Hsieh, Ming-Hsien Tsai, Jui-Wen Huang, Chih-Lung Lin, Hsiang-Wen Tseng, Zong-Keng Kuo, Jeng-Kai Jiang, Shung-Haur Yang, Chung-Wai Shiau, Kuen-Feng Chen

Supplement table 1.

General characteristics of colon cancer cohort (n=242)

**Characteristics**		**Number**	**%**
**Age (year-old)**	< 70	151	62.40
	≥ 70	91	37.60
**Location**	Left	142	58.68
	Right	100	41.32
**Initial clinical stage**	I	17	7.02
	II	67	27.69
	III	74	30.58
	IV	84	34.71
**Histopathologic subtype**	Adenocarcinoma	232	95.87
	Mucinous adenocarcinoma	9	3.72
	Signet ring cell carcinoma	1	0.41
**High histologic grade**		21	8.67
**Positive lymphovascular involvement**		55	22.73

Figure legends for supplement figures

Supplement Figure 1. PTP1B expression was significantly associated with the capability of colon sphere formation of Hct29 cell.

Hct29 cells with knockdown of PTP1B were seeded in ultra-low attachment 24-well plate and grew in median containing 10% FBS for 10 days.

Supplement Figure 2. PTP1B expression affects phospho-PITX1 via direct association

(A) PTP1B directly associated with PITX-1. The interactions between PTP1B and indicated proteins were detected by co-immunoprecipitation. CIP2A, an irrelevant protein, was used as negative control.

(B) Modulation of PTP1B levels influenced the phosphorylation status of endogenous PITX-1 in DLD1 cells. Ectopic expression of PTP1B induced tyrosine dephosphorylation of PITX-1 and silencing of PTP1B upregulated the phospho-PITX-1 level. Images of the triplicated experiments were shown here.

Supplement Figure 3. Degradation of PITX1 protein was promoted by PTP1B-induced dephosphorylation of PITX1.

(A, B) Specific PTP1B inhibitor treatment augmented PITX1/p120RasGAP expression in CRC cells. The dose- and time- dependent changes of the expression of PITX1/p120RasGAP after PTP1B treatment were also examined

by western blot. Images of triplicated dose-dependent experiments were shown here.

(C) PTP1B-dependent dephosphorylation reduced the stability of PITX-1. DLD1 cells with different PITX-1-phospho-mutants were treated with 50 µg/ml cycloheximide (CHX) for the indicated times and analyzed by western blot. Images of doublet experiments were shown over the left panel, and the results of quantified PITX1 protein expression were shown over the right panel.

Supplement Figure 4. Regorafenib upregulated PITX1/p120RasGAP expression by reducing proteasomal degradation of PITX1 protein.

(A, B) The dose- and time-dependent effects of regorafenib treatment on PITX1 and p120RasGAP expression were examined by western blot. Images and quantification results of the replicated results were shown here.

(C) Regorafenib treatment delayed the turnover of PITX1 protein. After exposure to 50 µg/ml cycloheximide (CHX) alone or co-treatment with regorafenib 5 µM at the indicated time, PITX1 protein expression in DLD1 cell were examined by western blot. Images of the triplicated experiments and quantification results were shown here.

(D) Regorafenib-induced augmentation of PITX1 expression was diminished in MG-231-treated cells. Effects of regorafenib on PITX1 protein expression in DLD1 and Hct-15 cells were determined after pretreated to MG-231, the proteasome inhibitor, or DMSO. Images of the triplicated experiments and

quantification results were shown here.

Supplement Figure 5.

(A) PTP1B expression is associated with the efficacy of regorafenib. Hct-29 cells with/without knockdown of PTP1B were exposed to regorafenib 2.5 μM for 24 hours and analyzed by MTT. Columns, mean; bars, SD (n=3); *, $P < 0.05$.

(B) The expressions of p-Erk in clinical CRC tumors were determined by immunohistochemical staining and the association of p-Erk and PTP1B were analyzed by Chi-square. Representative images of p-Erk in tumors were shown here. N=180.

Supplement Figure 1.

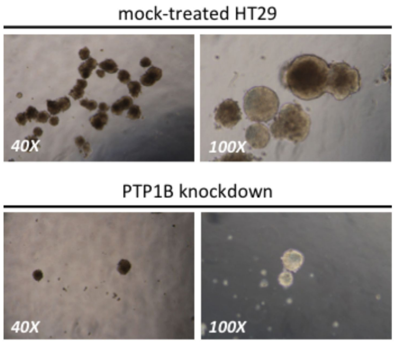

Supplement Figure 2.

(A)

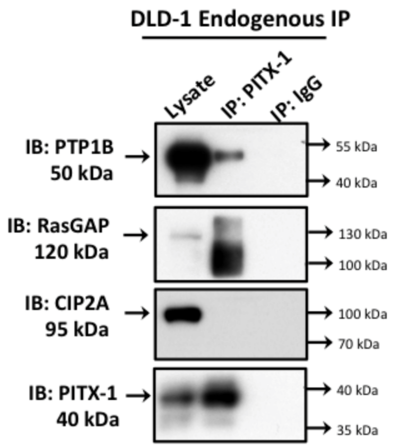

(B)

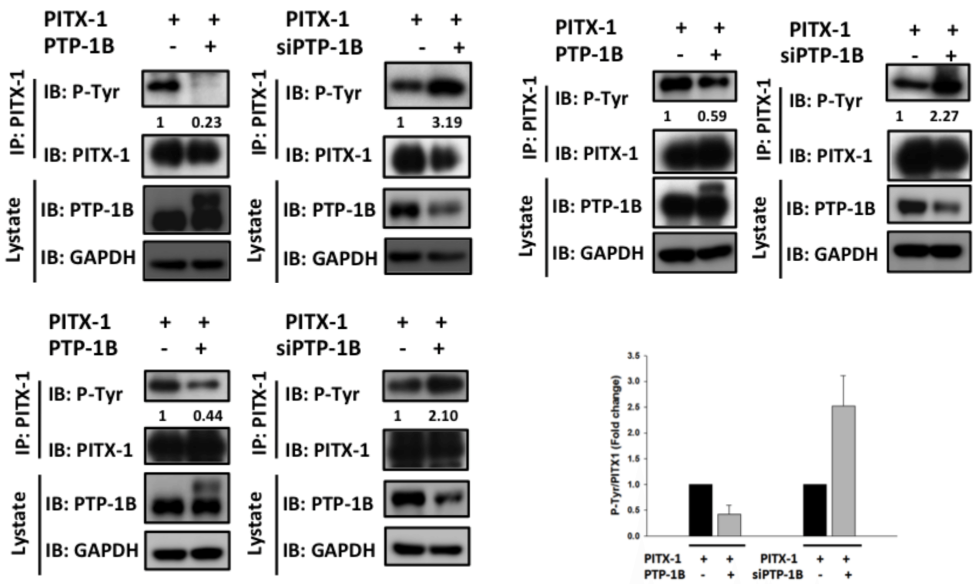

supplement Figure 3.

(A)

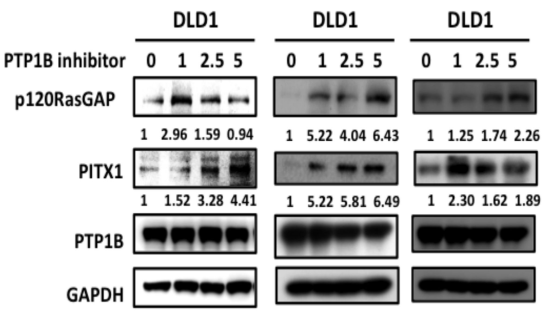

(B)

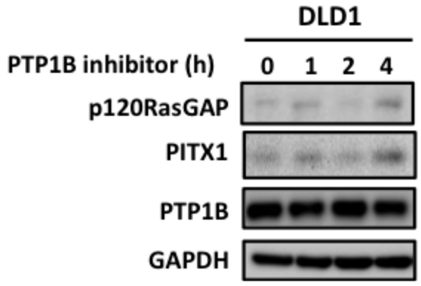

(C)

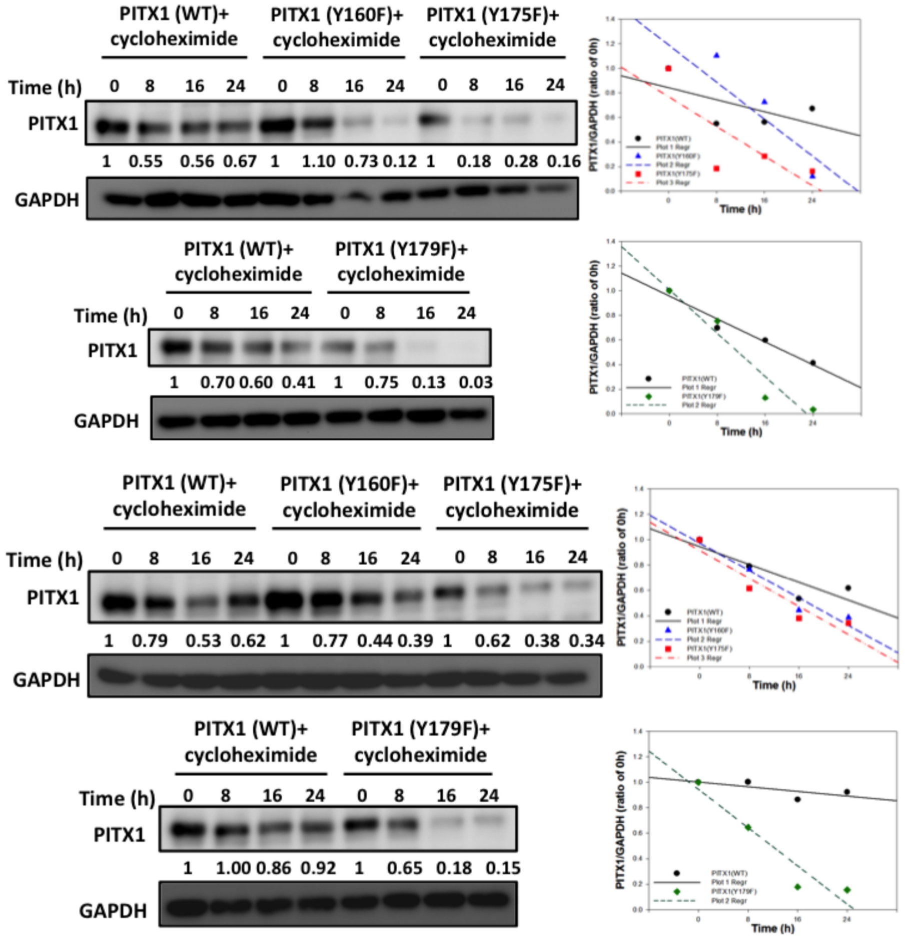

Supplement Figure 4.

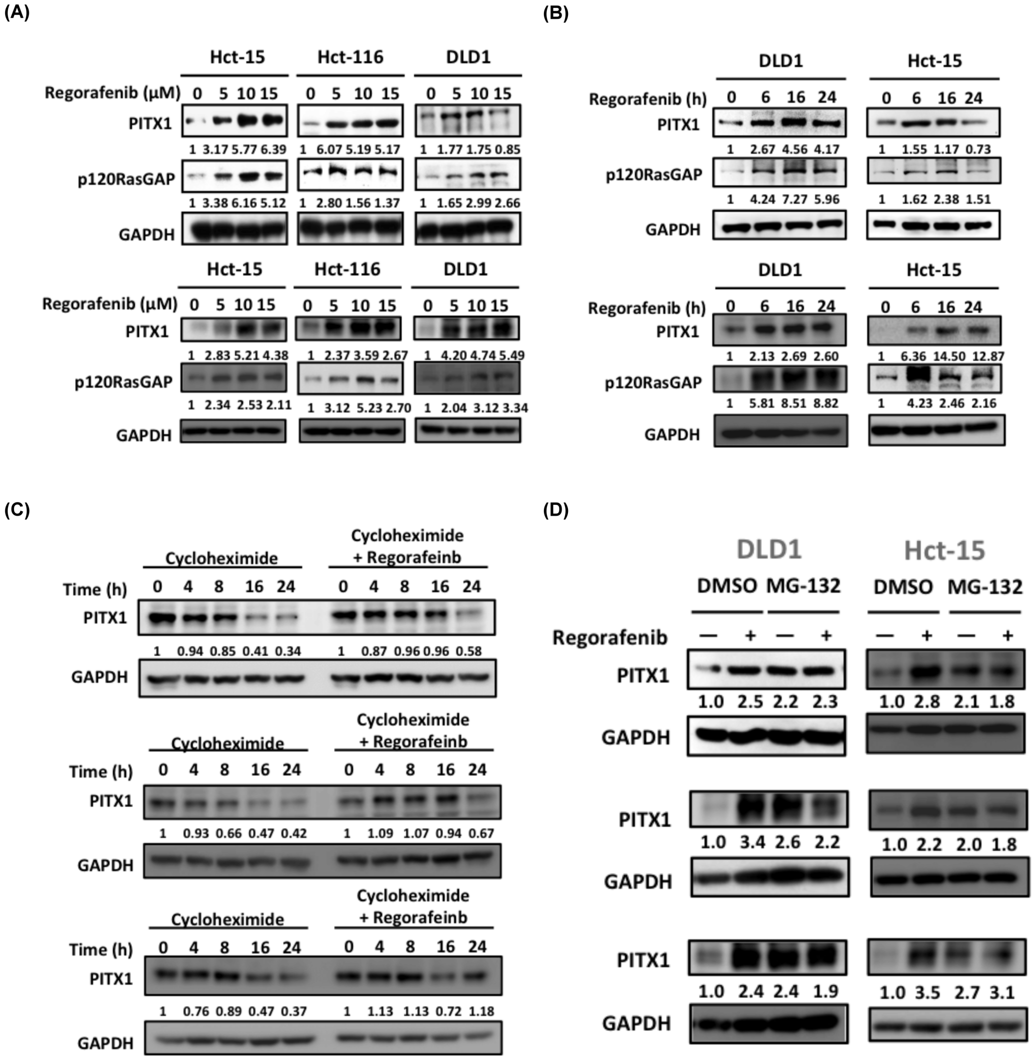

Supplement Figure 5.

(A)

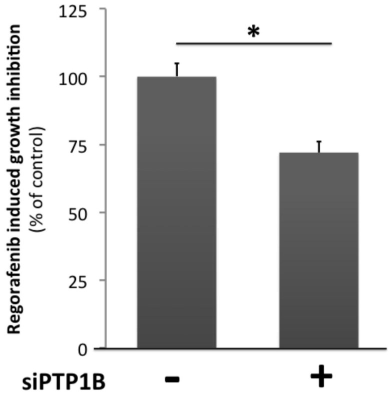

(B)

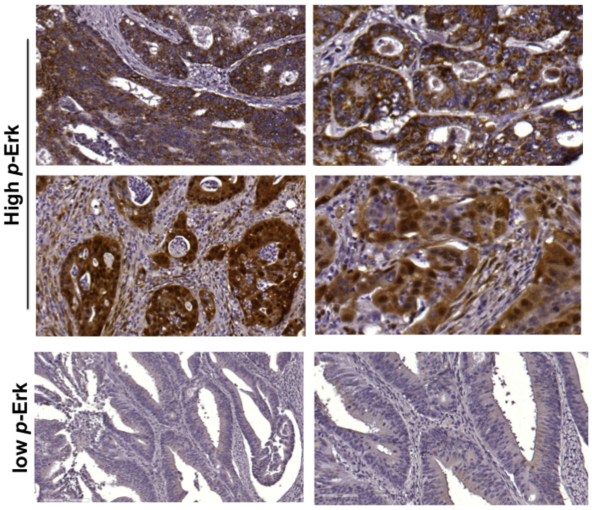

	PTP1B		
	Low	High	***p***
**High p-Erk**	29.7%	70.3%	0.001
**Low p-Erk**	55.2%	44.8%	
